# Exploring the Core Genes of Schizophrenia Based on Bioinformatics Analysis

**DOI:** 10.3390/genes13060967

**Published:** 2022-05-27

**Authors:** Shunkang Feng, Ping Sun, Chunhui Qu, Xiaohui Wu, Lu Yang, Tao Yang, Shuo Wang, Yiru Fang, Jun Chen

**Affiliations:** 1 Qingdao Medical College, Qingdao University, Qingdao 266071, China; fengshunkang@sina.com; 2 Qingdao Mental Health Center, Qingdao 266034, China; qdsunping99@sina.com (P.S.); qch4175@sina.com (C.Q.); 3 Clinical Research Center and Division of Mood Disorders, Shanghai Mental Health Center, Shanghai Jiao Tong University School of Medicine, Shanghai 200030, China; lindenwxh@163.com (X.W.); yangmedic0407@163.com (L.Y.); yangtaocomeon@163.com (T.Y.); wsshsmu@163.com (S.W.); 4 CAS Center for Excellence in Brain Science and Intelligence Technology, Shanghai 200031, China; 5 Shanghai Key Laboratory of Psychotic Disorders, Shanghai 201108, China

**Keywords:** schizophrenia, bioinformatics analysis, GEO

## Abstract

Schizophrenia is a clinical syndrome composed of a group of symptoms involving many obstacles such as perception, thinking, emotion, behavior, and the disharmony of mental activities. Schizophrenia is one of the top ten causes of disability globally, accounting for about 1% of the global population. Previous studies have shown that schizophrenia has solid genetic characteristics. However, the diagnosis of schizophrenia mainly depends on symptomatic manifestations, and no gene can be used as a clear diagnostic marker at present. This study explored the hub genes of schizophrenia by bioinformatics analysis. Three datasets were selected and downloaded from the GEO database (GSE53987, GSE21138, and GSE27383). GEO2R, NCBI’s online analysis tool, is used to screen out significant gene expression differences. The genes were functionally enriched by GO and KEGG enrichment analysis. On this basis, the hub genes were explored through Cytoscape software, and the immune infiltration analysis and diagnostic value of the screened hub genes were judged. Finally, four hub genes (NFKBIA, CDKN1A, BTG2, GADD45B) were screened. There was a significant correlation between two hub genes (NFKBIA, BTG2) and resting memory CD4 T cells. The ROC curve results showed that all four hub genes had diagnostic value.

## 1. Introduction

Schizophrenia is a common mental disease. More than 50% of the people diagnosed have intermittent but long-term mental problems, and about 20% have chronic symptoms and disabilities [1]. The life expectancy of patients is reduced by 10–20 years on average [2]. A study in China shows that the prevalence of schizophrenia is rising, from 0.39% in 1990 to 0.83% in 2010 [3]. It not only brings pain to patients but also places a serious burden on society. Another survey shows that schizophrenics cause an annual economic loss of GBP 11.8 billion to the British government [4].

At present, the diagnosis of schizophrenia mainly depends on symptomatic standards. However, because schizophrenia can have similar symptoms to other mental diseases, it is sometimes difficult to make an accurate diagnosis. Research on the genetic mechanism and diagnostic criteria of schizophrenia provides the possibility for an accurate diagnosis of schizophrenia. GEO (Gene Expression Omnibus, http://www.ncbi.nlm.nih.gov/geo, accessed on 24 May 2022) is an authoritative online database created and maintained by NCBI. The database contains multiple datasets of schizophrenia, including common species such as humans and mice [5]. At present, the bioinformatics analysis of schizophrenia mostly depends on the GEO database. In this study, three datasets with the GPL570 platform were screened through the GEO database. The tissue sources include the prefrontal cortex, striatum, hippocampus, and peripheral blood monocytes. The hub genes of schizophrenia were analyzed in bioinformatics. The common differentially expressed genes of the three datasets included 30 upregulated DEGs and 31 downregulated DEGs. GO and KEGG enrichment analysis showed that these DEGs were mainly enriched in nuclei, protein construction, and DNA-related biological processes. Four more reliable hub genes were screened out in the follow-up analysis process: NFKBIA, CDKN1A, BTG2, and GADD45B.

## 2. Materials and Methods

### 2.1. Data Source

The gene expression datasets analyzed in this study were all from the existing datasets from the GEO database. The data in the GEO database were processed before uploading to ensure that all data can be analyzed directly, and the disease group and control group of each dataset have obvious differentiation. This study aimed to conduct bioinformatics analysis, did not involve animals and human clinical experiments, and did not require ethical review. Because the clinical information in some datasets is incomplete, this study only took the platform and disease type as the screening criteria of the datasets. The three datasets included in this study are based on the GPL570[HG-U133_Plus_2] Affymetrix Human Genome U133 Plus 2.0 Array. The samples contained in GSE53987 were from the prefrontal cortex, striatum, and hippocampus of 19 patients with schizophrenia and 19 healthy individuals. Although GSE53987 contains different tissues, these different tissues are from the same group of experimental subjects. In this study, different tissues were regarded as a whole. Comparing the disease group with the control group can avoid the DEGs produced by different tissue sources, so as to screen the DEGs caused by the disease. The samples in GSE21138 were from the prefrontal cortex of 29 patients with schizophrenia and 30 healthy individuals. The samples in GSE27383 were peripheral blood mononuclear cell samples from 43 patients with schizophrenia and 29 healthy individuals.

### 2.2. Data Extraction for DEGs

All gene expression profiles were analyzed by the GEO2R online analysis tool. The GEO2R tool performs corrective analyses by default. There are significant differences in gene expression between patients with mental diseases and patients with other diseases (such as tumors). For example, if we use the differential expression screening criteria of tumor diseases, generally |log fold change (log FC)| > 1 or 2, there are few or even no differences in the DEGs of mental diseases that can be screened. Due to the particularity of mental illness, when the adj.P.value is used as the screening standard, the number of DEG generated will be too scarce for subsequent analysis, and some hub genes will be omitted. At this time, the P.value can be used as the screening standard to ensure the number of DEGs. In order to avoid missing more valuable DEGs, *p* < 0.05 and |log FC| > 0.1 were defined as the inclusion criteria of DEGs in this experiment. DEGs with log FC < 0 are considered gene downregulation, while DEGs with log FC > 0 are considered gene upregulation. Three datasets (GSE53987, GSE21138, and GSE27383) were screened for DEGs, and then the volcano map of the three datasets was drawn by using the volcano mapping tool (https://www.xiantao.love/products, accessed on 24 May 2022), and the intersection of up- and downregulated DEGs was screened by using the Venn map network tool (http://bioinformatics.psb.ugent.be/webtools/Venn/, accessed on 24 May 2022).

### 2.3. Functional Gene Ontology (GO) and KEGG Pathway Enrichment Analysis

DAVID (6.8) (https://david.ncifcrf, accessed on 24 May 2022) was used for GO and KEGG enrichment analysis of the screened DEGs and hub genes. GO includes three levels of analysis: biological process (BP), cellular component (CC), and molecular function (MF). KEGG is a common database for the study of genomes, biological pathways, diseases, chemicals, and drugs. In this study, when the GO annotation and KEGG enrichment pathway of DEGs meet *p* < 0.05 and count ≥ 4, they are of statistical value, while the hub gene meets *p* < 0.05. The bubble diagram was plotted by Weshengxin (http://www.bioinformatics.com.cn, accessed on 24 May 2022), a free online tool for data analysis and visualization.

### 2.4. Analysis of the Protein–Protein Interaction (PPI) Network and Hub Genes

The open online protein–protein interaction analysis tool STRING (https://string-db.org, accessed on 24 May 2022) was used to construct protein–protein interaction (PPI) network. The filtered DEGs were imported into the STRING database to evaluate PPI. Cytoscape software (https://cytoscape.org/, accessed on 24 May 2022) was used to generate a visual network of PPIs, and hub genes were jointly screened using CytoHubba and MCODE.

### 2.5. Gene–miRNA Analysis

Gene–miRNA interactions were analyzed using NetworkAnalyst (networkanalyst.ca, accessed on 24 May 2022) [6], and this tool was used to build a visual network.

### 2.6. Immune Infiltration Analysis

CIBERSORTx (stanford.edu) was used for immune infiltration analysis. Immune infiltration analysis was conducted combined with signature matrix for 22 types of immune cell composition of the prefrontal cortex of patients with schizophrenia and healthy people. R (version 3.6.3) and ggplot2 (version 3.3.3) were used to analyze the correlation between hub genes and different immune cells by the Pearson method and visualize the results. When *p* < 0.05, it was considered that there was a correlation between genes and immune cells.

### 2.7. Diagnostic Value of Hub Genes

An online receiver operating characteristic (ROC) curve drawing tool (https://www.xiantao.love/products, accessed on 24 May 2022) was used to help determine the diagnostic value of hub genes in schizophrenia. *p* < 0.05 means that the difference is statistically significant.

## 3. Results

### 3.1. Screening Differentially Expressed Genes (DEGs)

In this research, three microarray datasets (GSE53987, GSE21138, and GSE27383) were used to analyze the differential expression according to the criteria of *p* < 0.05 and |log FC| > 0.1. In the aggregate, 1091 differentially expressed genes (DEGs) were detected in GSE53987, of which 542 genes were upregulated and 549 genes were downregulated. In GSE21138, 3502 DEGs were identified, including 1833 upregulated genes and 1669 downregulated genes. In GSE27383, 3399 DEGs were identified, of which 1099 were upregulated and 2300 were downregulated (Table 1).

**Figure 1:**
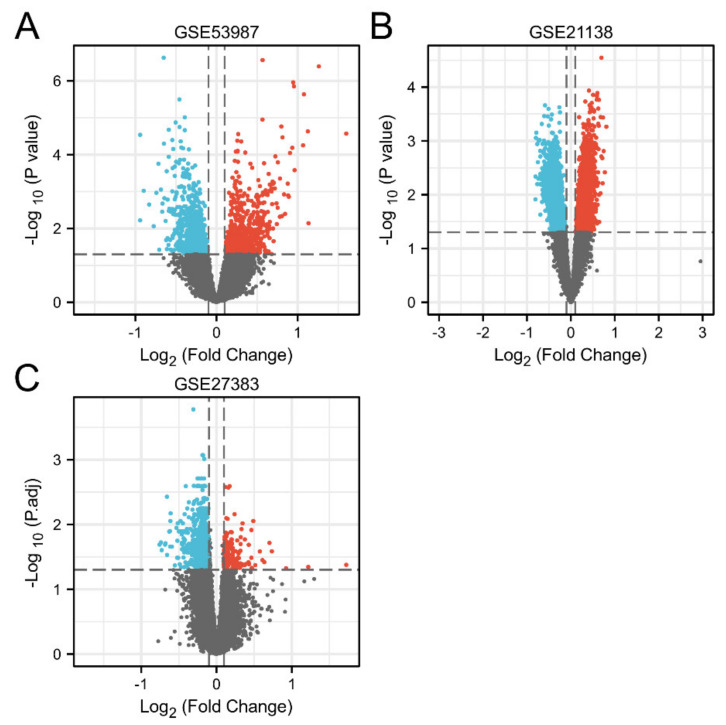
Screening DEGs between schizophrenia patients and healthy control individuals. (**A**) GSE53987; (**B**) GSE21138; (**C**) GSE27383.

The DEGs of three datasets (GSE53987, GSE21138, and GSE27383) of schizophrenia samples and healthy samples were visualized by a volcano map. In the volcanic map, DEGs between schizophrenia and healthy control individuals are represented by all nodes. In the figure, it is considered that there is a significant expression difference if *p* < 0.05 and |log FC| > 0.1. The genes with upregulated expression are marked in red, and the genes with downregulated expression are marked in blue. Volcano maps of GSE53987, GSE21138, and GSE27383 are shown in Figure 1A–C, respectively.

**Figure 2:**
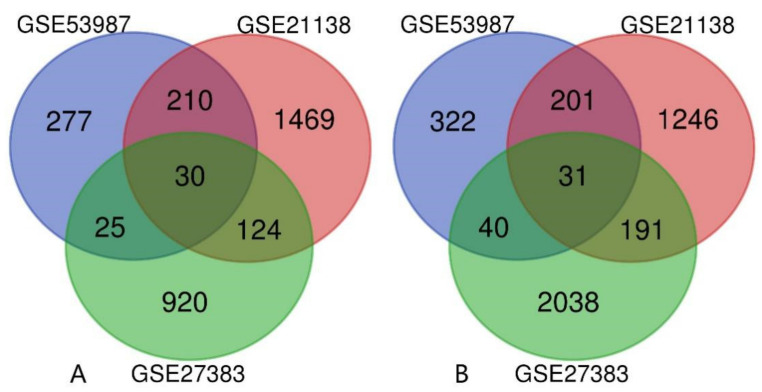
Venn diagram of DEGs from 3 GEO datasets. (**A**) Upregulated genes. (**B**). Downregulated genes.

Then, the intersection results of the three were obtained by Venn diagrams. As shown in Figure 2A,B, there are 61 DEGs in the intersection of the three datasets, of which 30 genes are upregulated and 31 genes are downregulated.

### 3.2. GO and KEGG Pathway Analysis of DEGs

**Figure 3:**
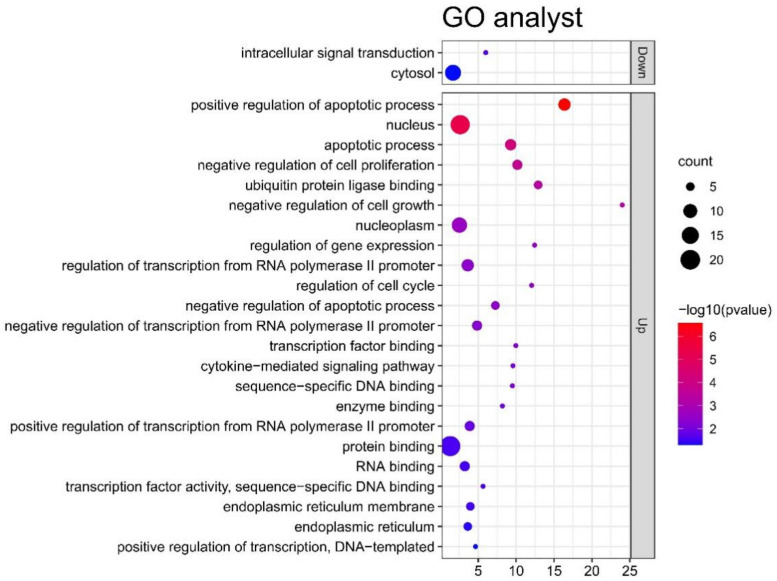
Significant GO terms of upregulated DEGs and downregulated DEGs.

According to the screening criteria of *p* < 0.05 and count ≥ 4, 30 upregulated genes and 31 downregulated genes were enriched and analyzed at three levels: biological process (BP), cell component (CC), and molecular function (MF). Figure 3 shows the results of the GO analysis. As shown in the figure, these genes participate in the pathogenesis of schizophrenia by participating in the corresponding functional pathways in the figure.

The GO analysis results show that upregulated DEGs are mainly enriched in protein binding, nucleus, nucleoplasm, and so on, and most of their functions are related to DNA, nucleus, and protein construction. The results of the GO analysis indicated that downregulated DEGs are enriched in the cytosol and intracellular signal transduction.

**Figure 4:**
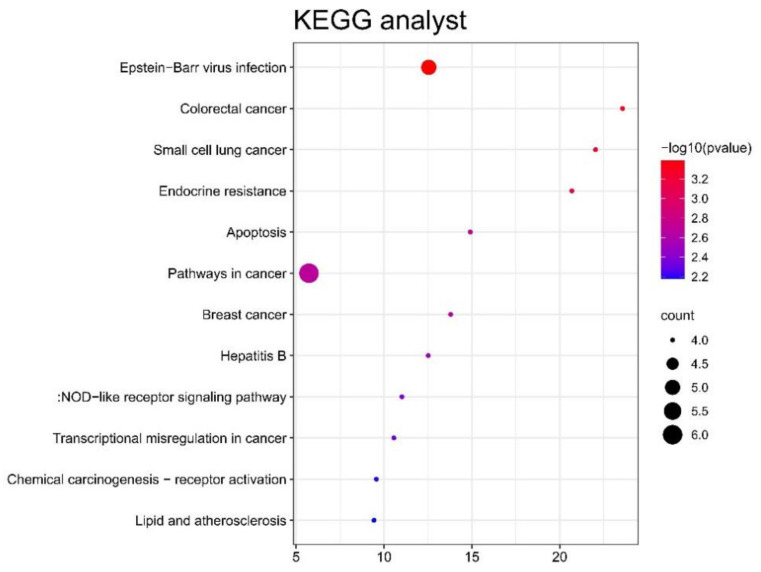
Significant KEGG pathways of upregulated DEGs.

Figure 4 shows the results of the KEGG pathway analysis of upregulated DEGs; they were mainly enriched in pathways in cancer, Epstein–Barr virus infection, colorectal cancer, small cell lung cancer, endocrine resistance, apoptosis, breast cancer, hepatitis B, NOD-like receptor signaling pathway, transcriptional misregulation in cancer, chemical carcinogenesis receptor activation, lipids, and atherosclerosis. However, the analysis of downregulated DEGs did not have meaningful results.

### 3.3. PPI Network Construction and Identification of Hub Genes

**Figure 5:**
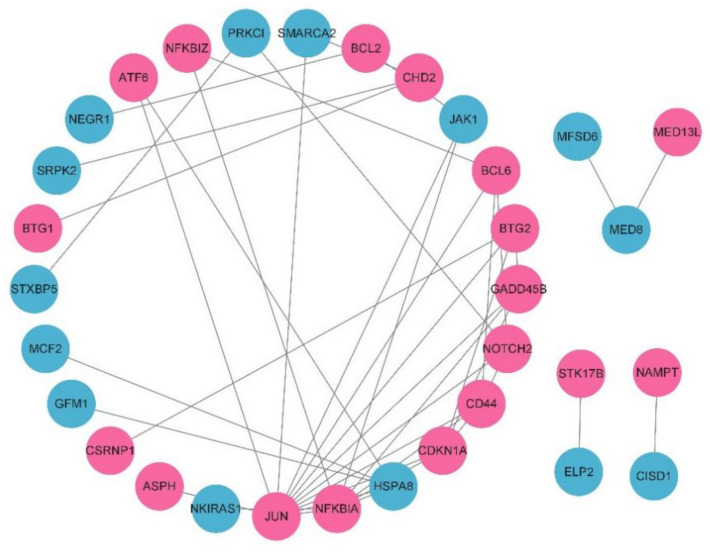
Protein–protein interaction network constructed with the DEGs. Red nodes represent upregulated genes, and blue nodes represent downregulated genes.

A PPI network was constructed by an online database string, and the results were imported into Cytoscape software (3.9.0) to further screen hub genes. PPI network information includes 58 nodes and 37 edges. After hiding disconnected nodes, 32 nodes and 42 edges remain. The results were imported into Cytoscape software (3.9.0) to build the network diagram, as shown in Figure 5.

**Figure 6:**
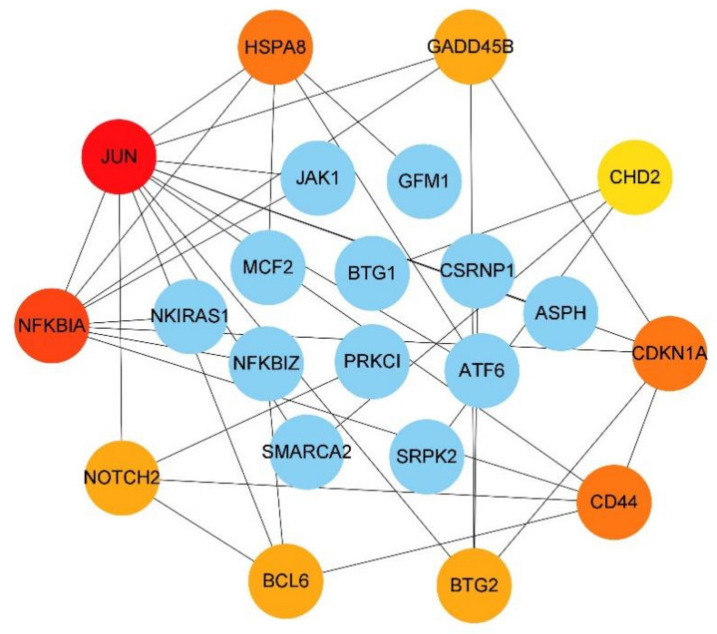
Protein–protein interaction network constructed with 10 hub genes and other DEGs. The darker the gene, the more prominent the core position in the network.

**Figure 7:**
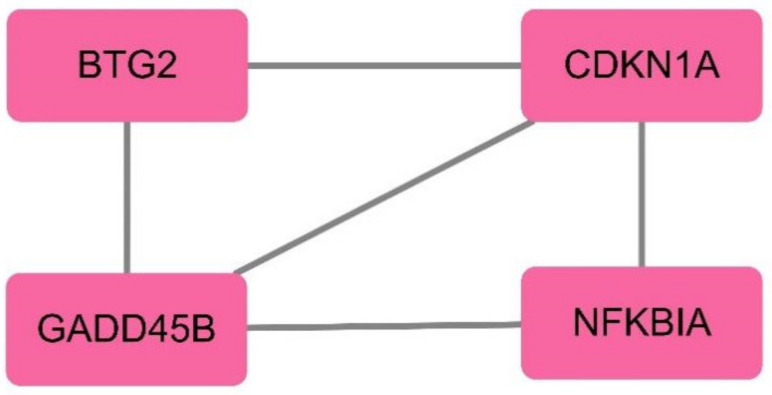
Cluster module extracted by MCODE.

Through CytoHubba, the top 10 genes were selected according to the degree ranking, as shown in Figure 6. A cluster module was extracted with the plug-in MCODE, as shown in Figure 7.

The intersection of the results of CytoHubba and MCODE was taken. The results include four hub genes: NFKBIA, CDKN1A, BTG2, and GADD45B. The expression of these hub genes is upregulated in schizophrenia samples (Table 2).

### 3.4. GO Analysis of Hub Genes

**Figure 8:**
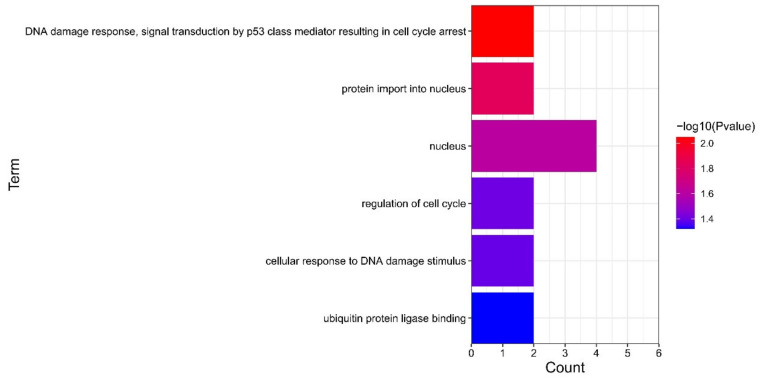
Significant GO terms of hub genes.

According to the screening standard of *p* < 0.05, GO enrichment analysis was carried out on the four screened hub genes, and the results are shown in Figure 8. Results of the GO analysis indicated that hub genes mainly enriched in DNA damage response, signal transduction by p53 class mediator resulting in cell cycle arrest, protein import into nucleus, nucleus, regulation of cell cycle, cellular response to DNA damage stimulus, and ubiquitin-protein ligase binding.

### 3.5. Gene–miRNA Analysis

**Figure 9:**
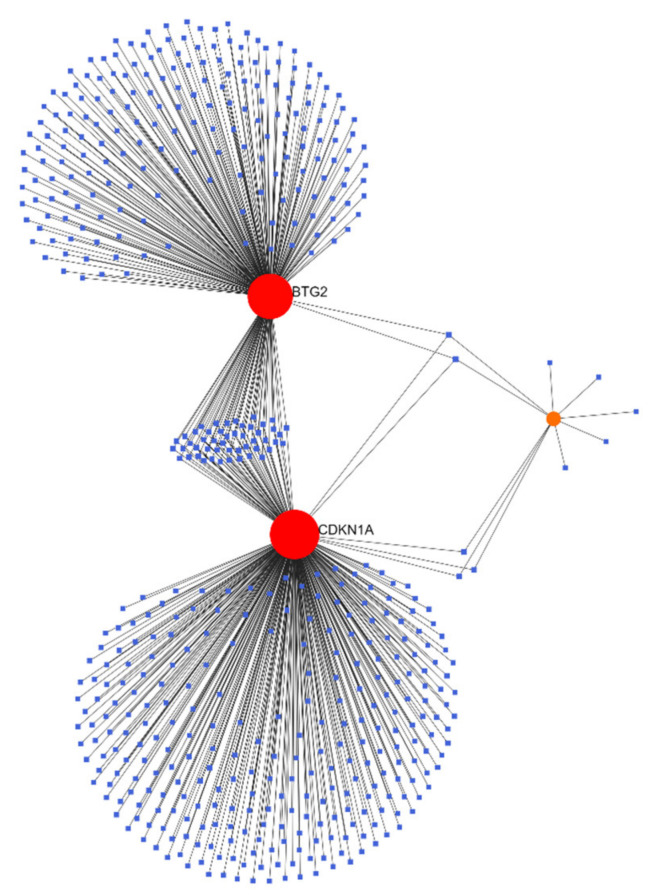
Predicted interactions among 3 core genes and their target miRNAs.

Gene–miRNA interactions were analyzed using NetworkAnalyst (networkanalyst.ca, accessed on 24 May 2022), and this tool was used to build a visual network. The results are shown in Figure 9.

A total of 532 target miRNAs of three hub genes and 588 mRNA-miRNA pairs were identified. According to the analysis results, NetworkAnalyst constructed an mRNA and miRNA coexpression network composed of 535 nodes and 588 lines. Predicted miRNAs and genes targeted by miRNAs can be seen in Table 3.

### 3.6. Immune Infiltration Analysis

**Figure 10:**
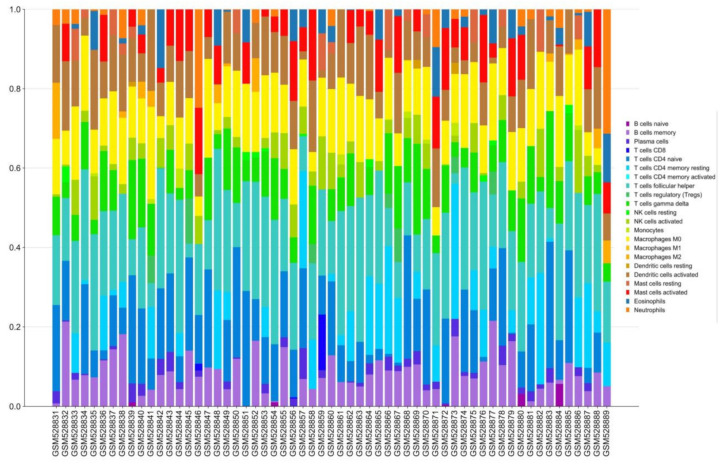
Immune infiltration analysis was conducted in combination with a signature matrix for 22 types of immune cell composition of the prefrontal cortex of patients with schizophrenia and healthy people.

The infiltration of 22 immune cells in brain tissue samples of 29 patients with schizophrenia and 30 healthy individuals was analyzed using the CIBERSORTx online analysis tool. The results are shown in Figure 10.

**Figure 11:**
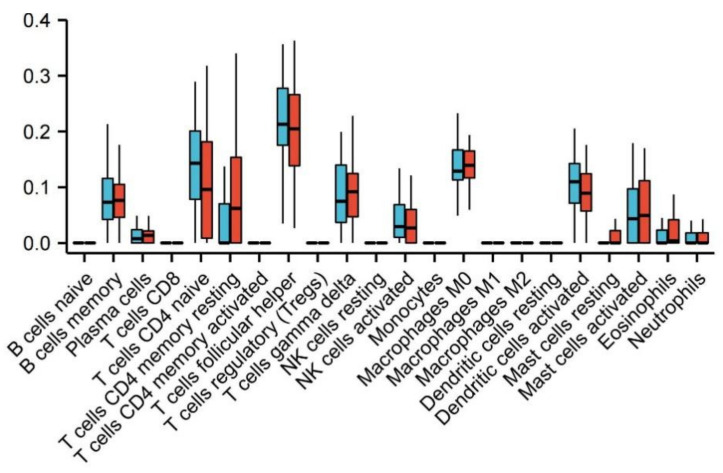
Comparison of immune cells in 22 patients with schizophrenia and healthy people. Blue represents healthy samples and red represents schizophrenic patients.

By comparing the composition of various immune cells between the schizophrenic group and the healthy group, it can be observed that there are significant differences in resting memory CD4 T cells in schizophrenic patients (Figure 11).

### 3.7. Correlation Analysis between Hub Genes and Resting Memory CD4 T Cells 

**Figure 12:**
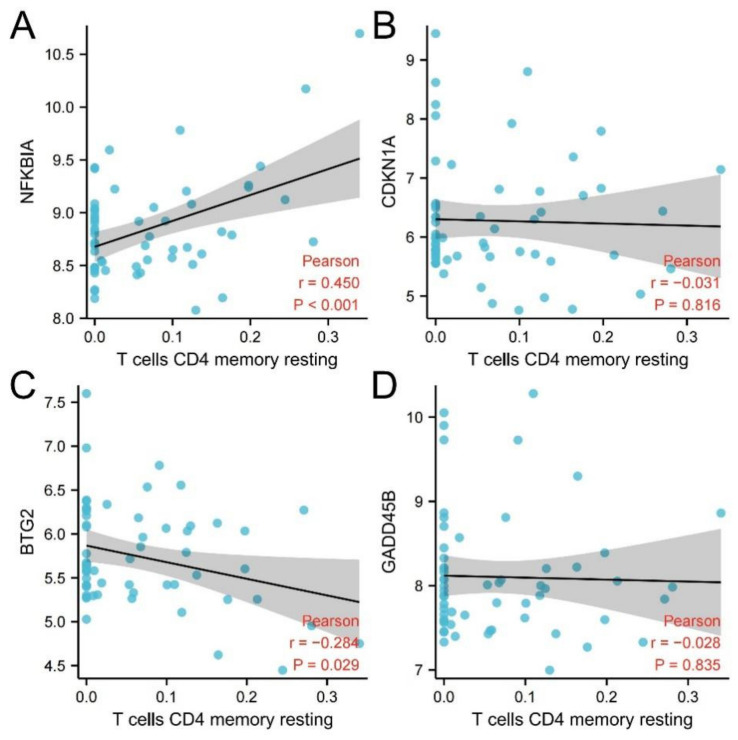
Correlation analysis between hub genes and resting memory CD4 T cells. (**A**) NFKBIA and resting memory CD4 T cells; (**B**) CDKN1A and resting memory CD4 T cells; (**C**) BTG2 and resting memory CD4 T cells; (**D**) GADD45B and resting memory CD4 T cells.

Then four hub genes and resting memory CD4 T cells were analyzed by R and the results were visualized through R. The results are shown in Table 4 and Figure 12.

### 3.8. Diagnostic Evaluation of Hub Genes

**Figure 13:**
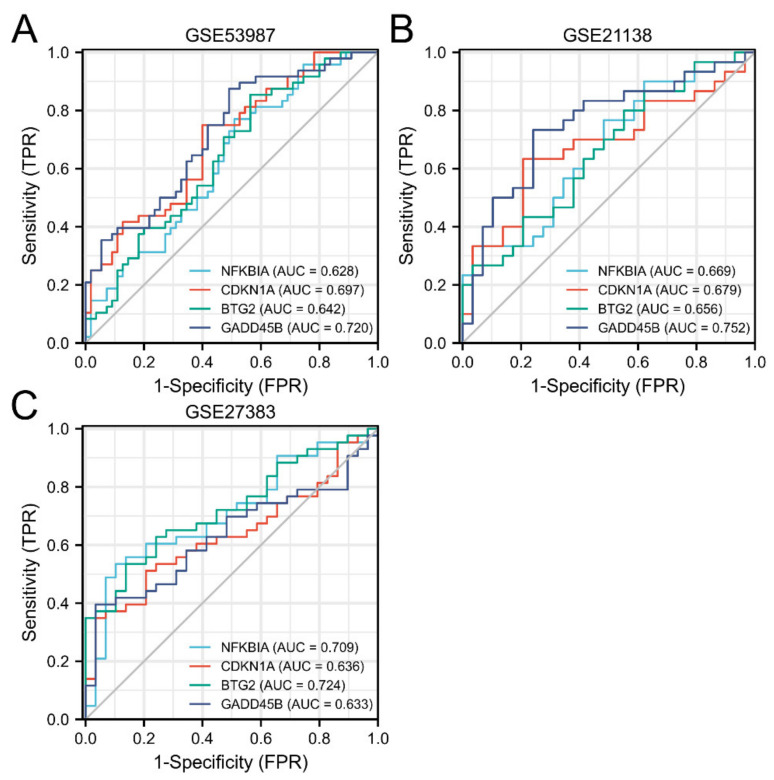
Diagnostic evaluation of 4 hub genes from three datasets. (**A**) GSE53987; (**B**) GSE21138; (**C**) GSE27383.

In order to further explore the diagnostic value of the four hub genes (NFKBIA, CDKN1A, BTG2, and GADD45B) for schizophrenia, this experiment used ROC curves for evaluation. As shown in Figure 13A, the area under the curve (AUC) values of 

NFKBIA, CDKN1A, BTG2, and GADD45B in schizophrenia and healthy samples measured in GSE53987 are 0.0628 (95% confidence interval (CI), 0.520–0.736), 0.697 (95% confidence interval (CI), 0.596–0.797), 0.642 (95% confidence interval (CI), 0.535–0.748), and 0.720 (95% confidence interval (CI), 0.622–0.818).

In Figure 13B, the AUC values of NFKBIA, CDKN1A, BTG2, and GADD45B in schizophrenia and healthy samples measured in GSE53987 are 0.669 (95% confidence interval (CI), 0.530–0.808), 0.679 (95% confidence interval (CI), 0.538–0.820), 0.656 (95% confidence interval (CI), 0.516–0.796), and 0.752 (95% confidence interval (CI), 0.624–0.880).

In Figure 13C, the AUC values for hub genes in the GSE27383 are 0.709 (95% confidence interval (CI), 0.587–0.831), 0.636 (95% confidence interval (CI), 0.508–0.764), 0.724 (95% confidence interval (CI), 0.609–0.840), and 0.633 (95% confidence interval (CI), 0.504–0.761).

In conclusion, four hub genes may have significant diagnostic value in the diagnosis of schizophrenia (AUC: 0.60–1.00).

**Figure 14:**
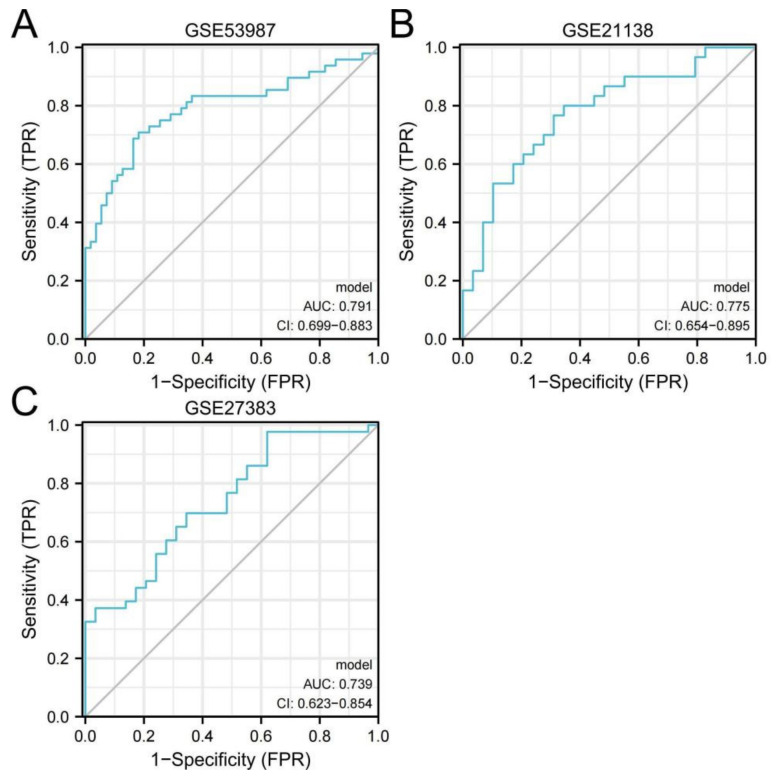
Diagnostic evaluation of joint indicators from three datasets. (**A**) GSE53987; (**B**) GSE21138; (**C**) GSE27383.

The results of considering the four hub genes as joint indicators to judge their diagnostic value are shown in Figure 14. In Figure 14A, the AUC value for the combination of the hub genes in GSE53987 is 0.791 (95% confidence interval (CI), 0.699–0.883). In Figure 14B, the AUC value for the combination of the hub genes in GSE21138 is 0.775 (95% confidence interval (CI), 0.654–0.895) In Figure 14C, the AUC value for the combination of the hub genes in GSE27383 is 0.739 (95% confidence interval (CI), 0.623–0.854). The results showed that the AUC of joint indicators was larger than that of a single hub gene, which suggested that when hub genes were regarded as a whole, they had higher diagnostic value and were more conducive to the judgment standard of schizophrenia in the clinic.

## 4. Discussion

Because of the disability it causes and its rising prevalence, schizophrenia causes not only serious damage to the health of patients but also incalculable economic losses to society. Therefore, the research on the pathogenesis and genetic mechanism of schizophrenia is of far-reaching significance. Genes with diagnostic value for schizophrenia can assist in the accurate diagnosis of schizophrenia, help reduce patients’ pain, and improve the prognosis of the disease.

NFKBIA (NFKB inhibitor α) is highly expressed in the bone marrow. Its encoded protein interacts with REL dimers to inhibit NF-kappa-B/REL complexes, participating in the regulation of inflammatory response [7].

The gene title of CDKN1A is cyclin-dependent kinase inhibitor 1a. The protein encoded by CDKN1A binds to and inhibits the activity of cyclin-dependent kinase 2 or cyclin-dependent kinase 4 complexes and thus functions as a regulator of cell cycle progression at G1 [7]. In previous studies, CDKN1A was found to be significantly downregulated in oligodendrocyte precursor cells and neurons in cortical cell cultures treated with quetiapine [8]. CDKN1A in three datasets is highly expressed in the schizophrenic population. In this study, quetiapine inhibited the expression level of CDKN1A, which can also be used to explain the therapeutic effect of quetiapine on schizophrenia.

The full gene name of BTG2 is BTG anti-proliferation factor 2. The encoded protein is involved in the regulation of G1/s conversion of the cell cycle [7].

GADD45B is growth arrest and DNA damage-inducible β. The transcript level of this gene increases under stress growth arrest conditions and after treatment with DNA-damaging agents, regulating the process of cell cycle or apoptosis [7]. In a previous study, it was found that neural activity will lead to the rapid expression of GADD45B, which is necessary for the promoter demethylation of key genes responsible for the development of newborn neurons, such as brain-derived neurotrophic factor (BDNF) [9]. The abnormal activation of GADD45B in the nervous tissue of schizophrenia is consistent with the high expression of GADD45B in the patient group in this study.

The GO enrichment analysis results of hub genes (NFKBIA, CDKN1A, BTG2, and GADD45B) also suggest that these genes are related to biological processes related to the nucleus, cell cycle, and DNA. As schizophrenia is a disease highly related to genetic factors, a better understanding of the mechanisms related to the nucleus and DNA is crucial in current research.

The interaction analysis between hub genes and mRNA revealed 532 directly related miRNAs, of which multiple miRNAs were related to three hub genes at the same time, which provided a new research idea for the action mechanism of subsequent genes. In the immune infiltration analysis, there was a significant difference in resting memory CD4 T cells between schizophrenia and healthy individuals. NFKBIA had a significant positive correlation with this cell type, while BTG2 had a negative correlation with it. Previous studies have found that inflammation plays a potential role in the pathogenesis and maintenance of schizophrenia [10], and the updated research also clearly shows that schizophrenia is related to the imbalance of immune response [11]. In this study, the number of resting memory CD4 T cells in schizophrenic patients was found to be significantly higher than that in healthy individuals, which may be related to the immune disorder of schizophrenic patients, and NFKBIA and BTG2 were significantly correlated with resting memory CD4 T cells, which further proves that the above two hub genes may be involved in the pathogenesis of schizophrenia. In the ROC curve, the AUC of the four hub genes in the three datasets was greater than 0.6. The results suggest that NFKBIA, CDKN1A, BTG2, and GADD45B have diagnostic value. The joint indicators of four hub genes have a more significant diagnostic value. In conclusion, NFKBIA, CDKN1A, BTG2, GADD45B, and the joint indicators of four hub genes have great potential as biomarkers and therapeutic targets for the diagnosis of schizophrenia.

However, there are still some limitations to this study. Firstly, this bioinformatics research on schizophrenia based on the GEO database is mainly based on the previous bioinformatics research strategies in the field of cancer. However, there are great differences between mental illness and cancer. If the previous analysis criteria for tumor diseases are applied to schizophrenia, not enough differentially expressed genes are screened to allow follow-up research. Usually, |log FC| uses 1 or 2 in the research of tumor diseases, but in this study, even if |log FC| is adjusted between 0.2 and 1, it is still unable to screen enough differentially expressed genes. However, if |log FC| is artificially set to 0.1, we can obtain enough DEGs for further analysis. This is the defect of the statistical screening standard in this study, but it is still widely used in the bioinformatics exploration of mental diseases. Second, although we found four hub genes, the mechanism of action of some of the four hub genes is not clear, and experiments have not been designed for further verification in this study. Adding relevant experiments for further verification should be the direction of future research.

## 5. Conclusions

Based on bioinformatics methods, this is the first study that found four hub genes closely related to schizophrenia (NFKBIA, CDKN1A, BTG2, and GADD45B). Enrichment analysis and immune infiltration analysis showed that the above genes might lead to the onset of schizophrenia by regulating the genetic process of cells or affecting the immune environment. However, considering the limitations of mental diseases in bioinformatics analysis, there are still some defects in applying this bioinformatics method based on the GEO database to mental diseases.

## Figures and Tables

**Table 1:** Statistical data for three GEO datasets (GSE53987, GSE21138, and GSE27383).

Datasets ID	Schizophrenia	Control	Patform	Sample Type	UP	Down
GSE53987	19	19	GPL570	Prefrontal cortex, striatum, and hippocampus	542	549
GSE21138	29	30	GPL570	Prefrontal cortex	1833	1669
GSE27383	43	29	GPL570	Peripheral blood mononuclear cells	1099	2300

**Table 2:** Results obtained by taking the intersection of the CytoHubba and MCODE.

Gene Symbol	logFC in GSE53987	logFC in GSE21138	logFC in GSE27383	Gene Title
NFKBIA	0.405603	0.289321	0.286481	NFKB inhibitor α
CDKN1A	0.630247	0.591038	0.237037	Cyclin-dependent kinase inhibitor 1A
BTG2	0.429461	0.224652	0.330094	BTG anti-proliferation factor 2
GADD45B	0.93334	0.498128	0.192576	Growth arrest and DNA damage-inducible β

**Table 3:** Predicted miRNAs and genes targeted by miRNAs.

miRNA	Genes Targeted by miRNA	Number of Related Hub Genes
hsa-let-7b-5p	BTG2, CDKN1A, NFKBIA	3
hsa-mir-93-5p	BTG2, CDKN1A, NFKBIA	3
hsa-mir-15a-5p	BTG2, CDKN1A	2
hsa-mir-16-5p	BTG2, CDKN1A	2
hsa-mir-17-5p	BTG2, CDKN1A	2
hsa-mir-20a-5p	BTG2, CDKN1A	2
hsa-mir-101-3p	BTG2, CDKN1A	2
hsa-mir-106a-5p	BTG2, CDKN1A	2
hsa-mir-15b-5p	BTG2, CDKN1A	2
hsa-mir-125a-5p	BTG2, CDKN1A	2
hsa-mir-195-5p	BTG2, CDKN1A	2
hsa-mir-106b-5p	BTG2, CDKN1A	2
hsa-mir-363-3p	BTG2, CDKN1A	2
hsa-mir-20b-5p	BTG2, CDKN1A	2
hsa-mir-519d-3p	BTG2, CDKN1A	2
hsa-mir-619-3p	BTG2, CDKN1A	2
hsa-mir-657	BTG2, CDKN1A	2
hsa-mir-149-3p	BTG2, CDKN1A	2
hsa-mir-505-5p	BTG2, CDKN1A	2
hsa-mir-665	BTG2, CDKN1A	2
hsa-mir-1207-5p	BTG2, CDKN1A	2
hsa-mir-1197	BTG2, CDKN1A	2
hsa-mir-544b	BTG2, CDKN1A	2
hsa-mir-1260b	BTG2, CDKN1A	2
hsa-mir-3202	BTG2, CDKN1A	2
hsa-mir-4254	BTG2, CDKN1A	2
hsa-mir-4267	BTG2, CDKN1A	2
hsa-mir-3659	BTG2, CDKN1A	2
hsa-mir-3663-3p	BTG2, CDKN1A	2
hsa-mir-3665	BTG2, CDKN1A	2
hsa-mir-3714	BTG2, CDKN1A	2
hsa-mir-3150b-3p	BTG2, CDKN1A	2
hsa-mir-4487	BTG2, CDKN1A	2
hsa-mir-4650-3p	BTG2, CDKN1A	2
hsa-mir-4728-5p	BTG2, CDKN1A	2
hsa-mir-4747-5p	BTG2, CDKN1A	2
hsa-mir-4763-3p	BTG2, CDKN1A	2
hsa-mir-4784	BTG2, CDKN1A	2
hsa-mir-5095	BTG2, CDKN1A	2
hsa-mir-5196-5p	BTG2, CDKN1A	2
hsa-mir-6511a-5p	BTG2, CDKN1A	2
hsa-mir-1910-3p	BTG2, CDKN1A	2
hsa-mir-6740-5p	BTG2, CDKN1A	2
hsa-mir-6753-5p	BTG2, CDKN1A	2
hsa-mir-6785-5p	BTG2, CDKN1A	2
hsa-mir-6798-5p	BTG2, CDKN1A	2
hsa-mir-6825-5p	BTG2, CDKN1A	2
hsa-mir-6854-5p	BTG2, CDKN1A	2
hsa-mir-6883-5p	BTG2, CDKN1A	2
hsa-mir-7151-3p	BTG2, CDKN1A	2
hsa-mir-203a-5p	BTG2, CDKN1A	2
hsa-mir-196a-5p	BTG2, CDKN1A	2
hsa-mir-335-5p	BTG2, CDKN1A	2
hsa-mir-942-5p	BTG2, CDKN1A	2

**Table 4:** Correlation analysis between hub genes and resting memory CD4 T cells.

Method	Cell	Gene	Correlation Coefficient	95% Confidence Interval	*p* Value
Pearson	Resting memory CD4 T cells	NFKBIA	r = 0.45	0.219–0.633	0.000353
Pearson	Resting memory CD4 T cells	CDKN1A	r = −0.031	−0.512–0.001	0.816
Pearson	Resting memory CD4 T cells	BTG2	r = −0.284	−0.473–0.081	0.029
Pearson	Resting memory CD4 T cells	GADD45B	r = −0.028	−0.512–0.001	0.835

## Data Availability

All data are obtained from public data in the GEO database.

## References

[1] Barbato A. (1998). Psychiatry in transition: Outcomes of mental health policy shift in Italy. Aust. N. Z. J. Psychiatry.

[2] Chesney E., Goodwin G.M., Fazel S. (2014). Risks of all-cause and suicide mortality in mental disorders: A meta-review. World Psychiatry.

[3] Chan K.Y., Zhao F.F., Meng S., Demaio A.R., Reed C., Theodoratou E., Campbell H., Wang W., Rudan I. (2015). Prevalence of schizophrenia in China between 1990 and 2010. J. Glob. Health.

[4] Commission S. (2012). The Abandoned Illness: A Report from the Schizophrenia Commission.

[5] Edgar R., Domrachev M., Lash A.E. (2002). Gene Expression Omnibus: NCBI gene expression and hybridization array data repository. Nucleic Acids Res..

[6] Zhou G., Soufan O., Ewald J., Hancock R.E.W., Basu N., Xia J. (2019). NetworkAnalyst 3.0: A visual analytics platform for comprehensive gene expression profiling and meta-analysis. Nucleic Acids Res..

[7] Fagerberg L., Hallström B.M., Oksvold P., Kampf C., Djureinovic D., Odeberg J., Habuka M., Tahmasebpoor S., Danielsson A., Edlund K. (2014). Analysis of the human tissue-specific expression by genome-wide integration of transcriptomics and antibody-based proteomics. Mol. Cell. Proteomics.

[8] Kondo M.A., Tajinda K., Colantuoni C., Hiyama H., Seshadri S., Huang B., Pou S., Furukori K., Hookway C., Jaaro-Peled H. (2013). Unique pharmacological actions of atypical neuroleptic quetiapine: Possible role in cell cycle/fate control. Transl. Psychiatry.

[9] Ma D.K., Jang M.H., Guo J.U., Kitabatake Y., Chang M.L., Pow-Anpongkul N., Flavell R.A., Lu B., Ming G.L., Song H. (2009). Neuronal activity-induced Gadd45b promotes epigenetic DNA demethylation and adult neurogenesis. Science.

[10] Müller N., Bechter K. (2013). The mild encephalitis concept for psychiatric disorders revisited in the light of current psychoneuroimmunological findings. Neurol. Psychiatry Brain Res..

[11] de Witte L., Tomasik J., Schwarz E., Guest P.C., Rahmoune H., Kahn R.S., Bahn S. (2014). Cytokine alterations in first-episode schizophrenia patients before and after antipsychotic treatment. Schizophr. Res..

